# Late‐Onset Crizotinib‐Associated Renal Cysts With Subsequent Regression Following Dose Reduction: A Case Report

**DOI:** 10.1111/1759-7714.70111

**Published:** 2025-06-21

**Authors:** Joon‐Young Yoon, Won Gi Jeong, Yoo‐Duk Choi, Seung Ji Kang, Hwa Kyung Park, Hyung‐Joo Oh, Cheol‐Kyu Park, In‐Jae Oh, Young‐Chul Kim

**Affiliations:** ^1^ Department of Internal Medicine, Division of Pulmonology Chonnam National University Medical School Hwasun Jeonnam South Korea; ^2^ Lung Cancer Center Chonnam National University Hwasun Hospital Hwasun Jeonnam South Korea; ^3^ Department of Radiology Chonnam National University Medical School Hwasun Jeonnam South Korea; ^4^ Department of Pathology Chonnam National University Medical School Gwangju South Korea; ^5^ Department of Internal Medicine, Division of Infectious Diseases Chonnam National University Medical School Hwasun Jeonnam South Korea

**Keywords:** adverse effects, carcinoma, Crizotinib, drug tapering, non‐small cell lung

## Abstract

Crizotinib, an anaplastic lymphoma kinase (ALK)/ROS1/c‐MET inhibitor, improves outcomes in ALK‐positive non‐small cell lung cancer (NSCLC) but can cause crizotinib‐associated renal cysts (CARCs), a rare yet clinically relevant adverse effect. We report a case of a 68‐year‐old Korean male who developed complex renal cysts after 4 years of crizotinib therapy. Radiologic findings initially raised suspicion for either an abscess or a neoplastic lesion, leading to surgical resection. However, recurrent renal cysts developed during continued crizotinib therapy, and CARCs were subsequently suspected. A dose reduction was implemented, which led to cyst regression without compromising tumor control. This case highlights the need to recognize and manage late‐onset toxicities during long‐term treatment, emphasizing the clinical value of multidisciplinary evaluation and tailored dose adjustments.

## Graphical abstract

- Renal cysts developed 4 years after starting crizotinib, representing a rare case of late‐onset Crizotinib Associated Renal Cysts (CARCs).
- Crizotinib dose reduction led to complete cyst regression without compromising tumor control.

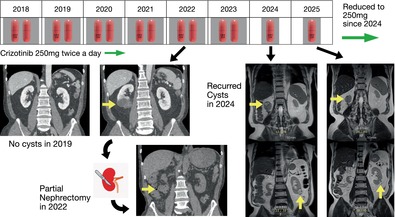

## 1 Introduction

Crizotinib is a first‐generation tyrosine kinase inhibitor (TKI) targeting anaplastic lymphoma kinase (ALK), ROS1, and c‐MET pathways [1]. Since its approval, it has revolutionized therapy for patients with ALK‐rearranged non‐small cell lung cancer (NSCLC), a subtype comprising 4%–7% of all NSCLC cases [2, 3]. Unlike conventional chemotherapy, crizotinib specifically inhibits oncogenic drivers, leading to significant improvements in progression‐free survival and overall response rates in ALK‐positive patients [4]. However, despite its targeted mechanism of action, crizotinib is associated with various side effects, ranging from common gastrointestinal symptoms to rarer toxicities such as crizotinib‐associated renal cysts (CARCs) [5, 6, 7, 8, 9].

Though rare, CARCs have been increasingly reported in clinical settings. These cysts can range from simple, asymptomatic lesions to complex, symptomatic ones requiring intervention. Since these adverse effects can mimic malignancy or infection, they may lead to unnecessary diagnostic or therapeutic procedures [5, 10, 11, 12].

Here, we report a case of a 68‐year‐old Korean male with ALK‐positive lung adenocarcinoma who developed CARCs during long‐term therapy. This case underscores the importance of regular monitoring, accurate diagnosis, multidisciplinary consultations, and individualized management, including dose adjustment to mitigate drug‐related complications while maintaining optimal oncologic outcomes.

This case report was approved by the Institutional Review Board of Chonnam National University Hwasun Hospital (CNUHH‐2025‐083), and written informed consent was obtained from the patient using the approved consent form.

### 1.1 Case Presentation

**FIGURE 1:**
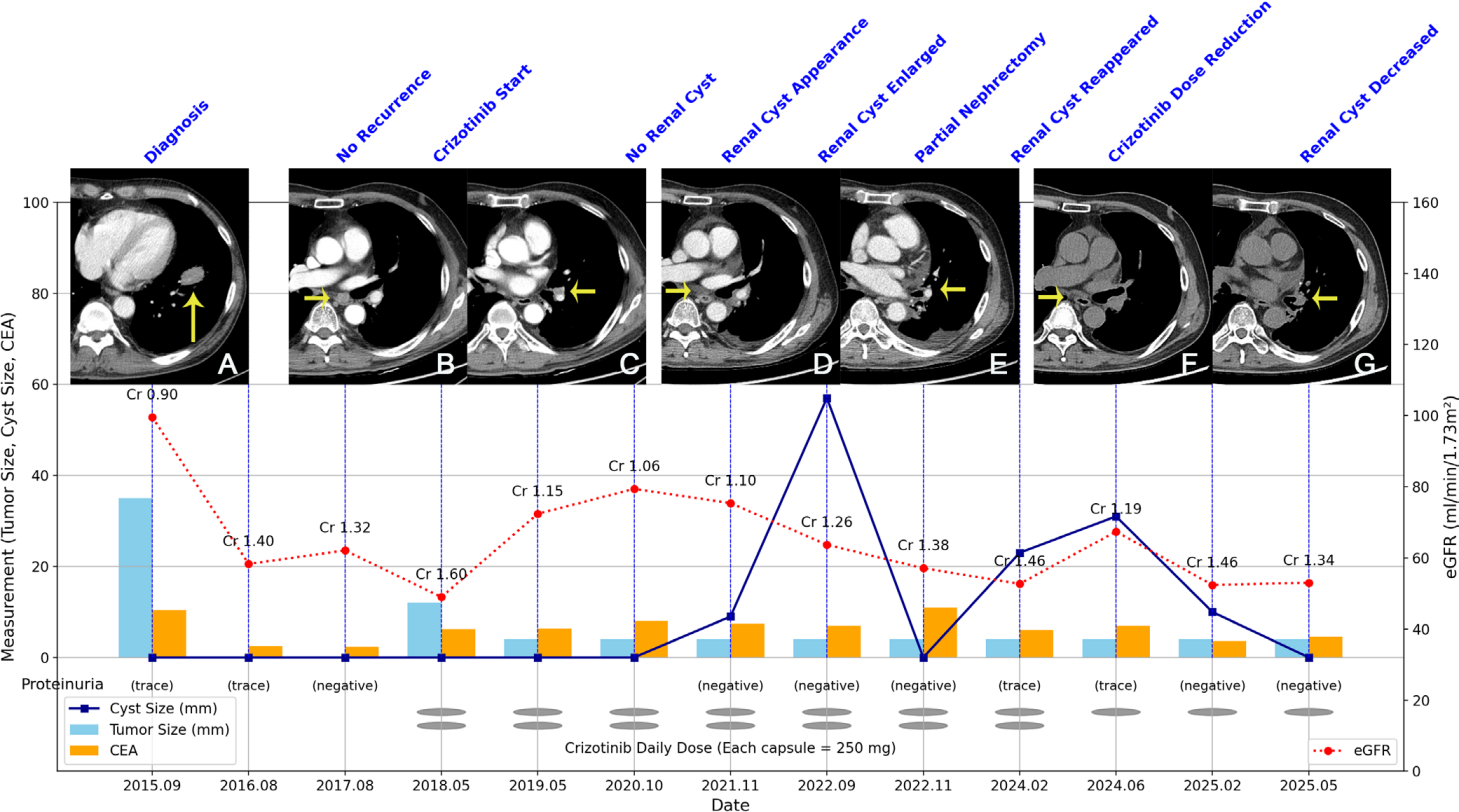
Clinical course and tumor response in a patient with ALK‐positive lung adenocarcinoma treated with Crizotinib. The graph summarizes serial changes in tumor size, cyst size, carcinoembryonic antigen (CEA), and estimated glomerular filtration rate (eGFR), along with serum creatinine levels (Cr) indicated numerically. Crizotinib dosing is illustrated using gray capsule icons (each representing 250 mg), and proteinuria status is indicated below the *x*‐axis. Key clinical events are annotated above the graph. Representative axial CT images of the primary lung tumor are shown at selected time points (yellow arrows), demonstrating initial diagnosis (A), progression (B, C), partial response (D, E), and sustained control of the primary lesion during crizotinib treatment in reduced dose (F, G). The appearance, enlargement, and regression of renal cysts are temporally aligned with crizotinib exposure and dose adjustment.

In September 2015, a 58‐year‐old Korean male with a 33 pack‐year smoking history, who had quit smoking 5 years earlier, presented with an incidentally detected lung mass in the left lower lobe (Figure 1A). A staging workup revealed no evidence of distant metastasis.

In October 2015, he underwent a wedge resection of the lung using video‐assisted thoracoscopic surgery, which revealed ALK‐rearranged lung adenocarcinoma (pT4N0M0). Fluorescence in situ hybridization (FISH) testing with the Vysis LSI Dual Color Break Apart Probe Kit confirmed ALK gene translocation in more than 15% of tumor cells. Due to incomplete resection, the patient underwent concurrent chemoradiotherapy (6000 cGy).

In April 2018, a follow‐up computed tomography (CT) scan revealed an enlargement of the left lower mediastinal and interlobar lymph nodes (Figure 1B,C). Endobronchial ultrasound‐guided transbronchial needle aspiration (EBUS‐TBNA) confirmed recurrent adenocarcinoma, As a result, crizotinib therapy (250 mg twice daily) was initiated in May 2018, leading to partial remission (Figure 1D,E). In October 2019, additional radiotherapy (5000 cGy) was administered to address oligoprogression.

**FIGURE 2:**
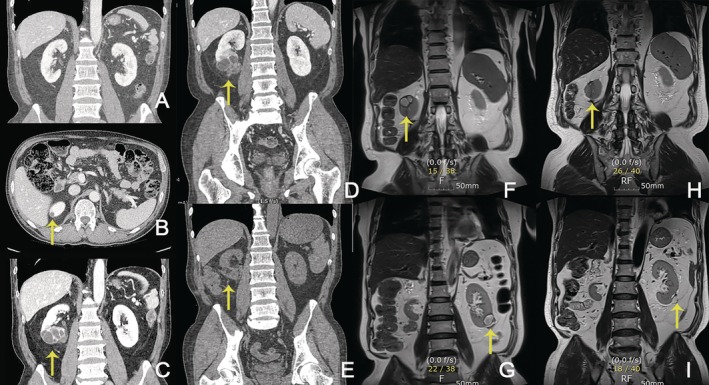
Serial abdominal computed tomography (CT) and magnetic resonance imaging (MRI) findings. (A) No renal cysts were observed 15 months after the initiation of crizotinib (August 2019). (B) A small cystic lesion in the upper pole of the right kidney was first detected on the abdominal slices of a chest CT scan 42 months after the initiation of crizotinib treatment (November 2021). (C) A newly developed complex cystic lesion in the lower pole of the right kidney, detected 49 months after the initiation of crizotinib treatment (June 2022). (D) An increase in the size of the complex renal cysts was noted 52 months after crizotinib initiation (September 2022). (E) Postoperative CT scan following partial nephrectomy (November 2022). (F, G) Enlargement of renal cysts in both kidneys (May 2024). (H, I) Complete remission of the renal cyst was observed after crizotinib dose reduction (May 2025).

**FIGURE 3:**
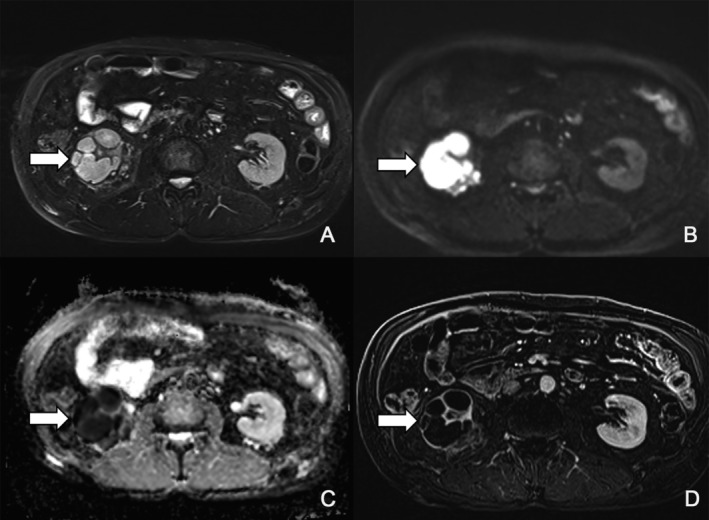
Renal Magnetic resonance imaging (MRI) findings. (A) An axial T2‐weighted fat‐suppressed image shows a multiseptated mass in the right kidney with heterogeneous high signal intensity. (B, C) The mass exhibits diffusion restriction on an axial diffusion‐weighted image (DWI) (*b*‐value = 1000 s/mm^2^) and the corresponding apparent diffusion coefficient (ADC) map. (D) The mass demonstrates thick septal and wall enhancement on an axial postcontrast subtraction image acquired at 60 s.

Until August 2019, no renal cysts were observed on abdominal CT (Figure 2A). However, in November 2021, a small cyst was identified in the upper pole of the right kidney on the abdominal slices of a chest CT scan (Figure 2B). In June 2022, an abdominal CT revealed a newly detected 3.7 cm amorphous enhancing lesion with adjacent caliectasis in the lower pole of the right kidney (Figure 2C). In July 2022, an magnetic resonance imaging (MRI) showed a 5.7 cm multiseptated cystic mass in the right kidney's lower pole with heterogeneous T2 hyperintensity, some T1 and T2 hyperintense areas, and diffusion restriction. Mild perirenal fat plane stranding was also noted, along with a 1.8 cm cystic lesion in the upper pole of the right kidney (Figure 3).

**FIGURE 4:**
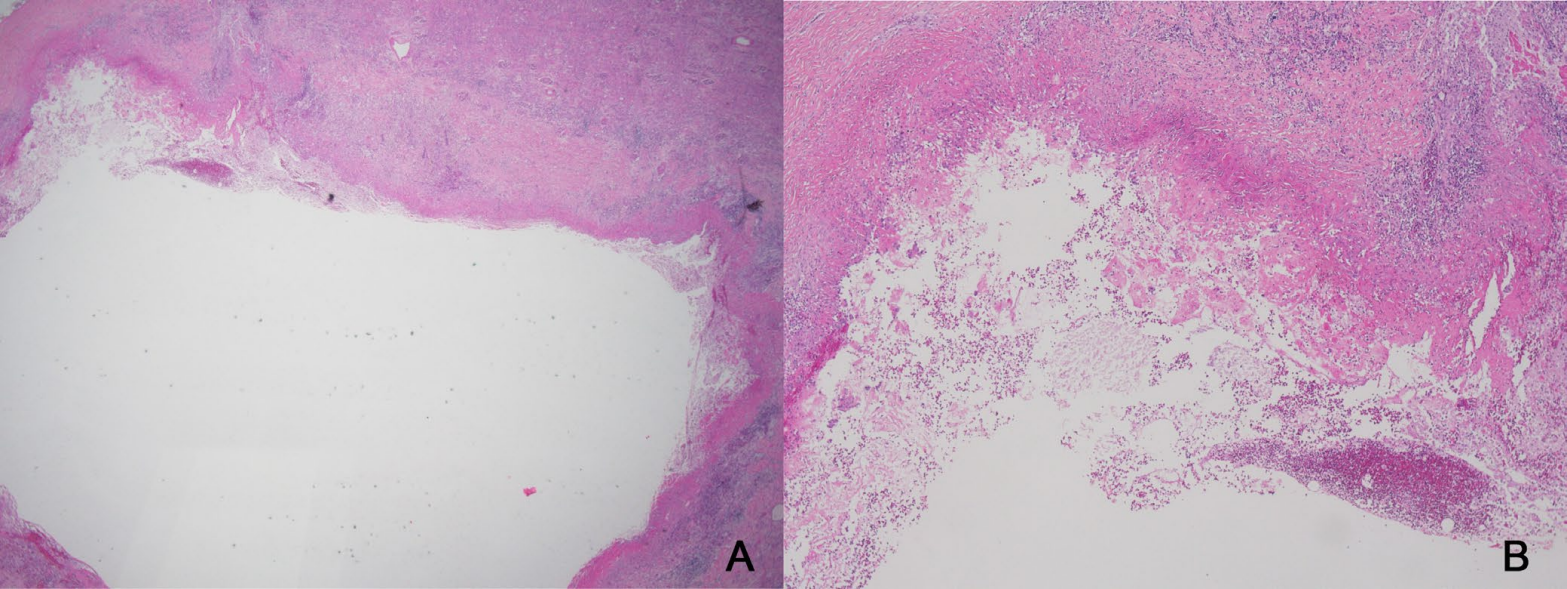
Pathologic findings after hematoxylin and eosin staining. (A) Inflammatory cystic lesion within the renal parenchyma (X10). (B) Mixed inflammatory cells with fibrinoid necrotic changes in the fibrous capsule of the cyst (X100)

By September 2022, a follow‐up CT showed that the lesion had increased in size to 4.3 cm, raising concern for malignancy (Figure 2D). Urinalysis showed no hematuria, and urine cytology was negative. Based on a multidisciplinary discussion involving the prescribing physician, radiologist, and urologist, surgical resection and biopsy were planned to differentiate between a hemorrhagic abscess and a neoplastic lesion. In November 2022, the patient underwent partial nephrectomy (Figure 2E), and histopathology confirmed an old abscess with fibrous capsule formation (Figure 4).

Crizotinib was continued after nephrectomy until February 2024, when enlargement of cysts in both the right and left kidneys was observed (Figure 2F,G). Since the renal biopsy pathology had revealed an old abscess, the prescribing physician conducted a follow‐up multidisciplinary consultation with a radiologist, urologist, and infectious disease specialist. This led to the suggestion that the lesions could represent CARCs. Although CARCs were considered a possible adverse effect of crizotinib, the causal relationship was not definitive. As the patient maintained a partial response without evidence of disease progression, switching to a second‐generation ALK TKI could have posed reimbursement challenges. Therefore, dose reduction was chosen as the initial management strategy.

There are currently no specific dose reduction guidelines for CARC. We followed the general dosing recommendations provided in the drug information, which suggest a two‐step reduction from 250 mg twice daily to 200 mg twice daily, and subsequently to 250 mg once daily. However, given the patient's decreased renal function, we opted to reduce the dose directly from 250 mg twice daily to 250 mg once daily, starting in June 2024.

By May 2025, follow‐up imaging demonstrated significant regression of the renal cysts without tumor progression, indicating a sustained anti‐tumor effect of reduced crizotinib dose (Figure 1F,G). A subsequent renal MRI confirmed a complete remission of the cysts (Figure 2H,I).

## 2 Discussion

CARCs have been reported in approximately 4% of patients treated with crizotinib [13]. Their pathogenesis remains unclear, but proposed mechanisms involve the inhibition of c‐MET, which plays a role in cellular repair and regeneration [14]. These cysts are hypothesized to result from off‐target effects on renal tubular cells, potentially mediated via the c‐MET pathway [8]. c‐MET receptors are expressed in renal tubular epithelium [15], and their inhibition may lead to the dysregulation of tubular function, resulting in cyst formation. In this case, a reduction in crizotinib dosage led to regression of renal cysts, supporting the role of crizotinib in cystogenesis and highlighting the potential reversibility of this adverse effect [7]. To date, there have been no reports of renal cyst development associated with next generation AKL inhibitors [16]. In contrast, several cases have been reported in which CARCs regressed following a switch from crizotinib to alectinib [17, 18]. Unlike crizotinib, alectinib does not inhibit c‐MET, which is thought to play a central role in the pathogenesis of CARCs.

Radiologic features of CARCs include multiloculated cystic lesions with enhancing walls or septa [13]. MRI provides detailed characterization, including diffusion restriction and signal intensity variations [19]. Recognizing these features is essential to avoid misdiagnosis as renal cell carcinoma or abscesses, which may lead to unnecessary interventions. Routine lung cancer follow‐up CT scans often exclude the lower kidney, potentially missing renal complications. In our case, only chest CT scans were performed during follow‐up, which may have delayed the detection of the renal cyst located in the lower pole of the kidney. Given the potentially higher CARCs risk in Asians, particularly Koreans [5], clinicians should consider regular abdominal imaging in high‐risk patients for early detection.

In our case, complex renal cysts developed after 4 years of treatment, significantly later than the typical onset of CARCs which usually occurs within a few months of therapy [5]. To the best of our knowledge, no previous reports have documented such a late occurrence of CARCs, emphasizing the need for long‐term monitoring. We considered the possibility that secondary triggers, such as infections, dehydration, or drug interactions, might have contributed to the delayed onset of the renal cysts. However, after a thorough review of the patient's clinical history, no new medications or clinically significant events were identified.

Biopsy specimens from CARCs typically reveal fibrous capsules with chronic inflammatory infiltrates, as seen in this case. The absence of neoplastic cells and the presence of nonspecific findings, such as granulation tissue, are hallmarks of CARCs [5, 11, 12, 18]. However, invasive procedures carry risks and should be balanced against the likelihood of CARCs.

Although spontaneous regression of CARCs has been reported [20], adjusting the crizotinib dosage represents a more effective strategy to mitigate adverse effects while maintaining therapeutic efficacy [5]. In this case, the radiologic features and clinical context suggested CARCs, prompting the multidisciplinary team to recommend a dose reduction rather than further invasive diagnostics. The dose reduction led to significant cyst shrinkage without compromising oncologic control, highlighting the potential for individualized treatment. This approach emphasizes the importance of integrating imaging findings with clinical judgment to refine diagnostic strategies and improve patient safety and treatment efficacy.

## Author Contributions

All authors had full access to the data in the study and take responsibility for the integrity of the data and the accuracy of the data analysis. Conceptualization: Y.‐C.K. and J.‐Y.Y. Data curation: I.‐J.O., C.‐K.P., H.‐J.O., and H.K.P. Resources: Y.‐D.C., S.J.K., and W.G.J. Supervision: Y.‐C.K. Writing – original draft: Y.‐C.K. and J.‐Y.Y. Writing – review and editing: Y.‐C.K. I.‐J.O., C.‐K.P., H.‐J.O., H.K.P., and J.‐Y.Y.

## Conflicts of Interest

The authors declare no conflicts of interest.

## Data Availability

The data that support the findings of this study are available from the corresponding author upon reasonable request.
